# Free abdominal fluid without obvious solid organ injury upon CT imaging: an actual problem or simply over-diagnosing?

**DOI:** 10.1186/1752-2897-3-10

**Published:** 2009-12-15

**Authors:** Vanessa M Banz, Muhammad U Butt, Heinz Zimmermann, Victor Jeger, Aristomenis K Exadaktylos

**Affiliations:** 1 Visceral Surgery and Medicine, Inselspital, Berne, University Hospital and University of Berne, Switzerland; 2 Trauma, Emergency Surgery, Surgical Critical Care, MGH, Harvard Medical School, Boston, Massachusetts, USA; 3 Academic Emergency Medicine, Inselspital, Berne, University Hospital and University of Berne, Switzerland

## Abstract

Whereas a non-operative approach for hemodynamically stable patients with free intraabdominal fluid in the presence of solid organ injury is generally accepted, the presence of free fluid in the abdomen without evidence of solid organ injury not only presents a challenge for the treating emergency physician but also for the surgeon in charge. Despite recent advances in imaging modalities, with multi-detector computed tomography (CT) (with or without contrast agent) usually the imaging method of choice, diagnosis and interpretation of the results remains difficult. While some studies conclude that CT is highly accurate and relatively specific at diagnosing mesenteric and hollow viscus injury, others studies deem CT to be unreliable. These differences may in part be due to the experience and the interpretation of the radiologist and/or the treating physician or surgeon.

A search of the literature has made it apparent that there is no straightforward answer to the question what to do with patients with free intraabdominal fluid on CT scanning but without signs of solid organ injury. In hemodynamically unstable patients, free intraabdominal fluid in the absence of solid organ injury usually mandates immediate surgical intervention. For patients with blunt abdominal trauma and more than just a trace of free intraabdominal fluid or for patients with signs of peritonitis, the threshold for a surgical exploration - preferably by a laparoscopic approach - should be low. Based on the available information, we aim to provide the reader with an overview of the current literature with specific emphasis on diagnostic and therapeutic approaches to this problem and suggest a possible algorithm, which might help with the adequate treatment of such patients.

## Review

The introduction of routine computed tomography (CT) in trauma exposes us to a plethora of new information, sometimes leaving us with more information than we had bargained for. Although a recent study by Huber-Wagner and colleagues was able to show a positive effect on overall survival of trauma patients with blunt injury receiving whole-body CT during emergency department resuscitation [1], the study does not specifically evaluate abdominal trauma and free intraabdominal fluid without solid organ injury. The question as to what to do with this subgroup of patients remains a matter of debate.

Whilst sonography and conventional radiography remain well-established techniques, CT scanning of the abdomen and pelvis is the procedure of choice to evaluate the hemodynamically stable patient who has sustained blunt or penetrating trauma. CT has replaced Diagnostic Peritoneal Lavage (DPL) as the first method of choice in many trauma centers worldwide. Its major advantage is that it is not only capable of revealing the presence of intra-abdominal or intra-thoracic hemorrhage but can to some extent also identify the organ involved [2].

CT exhibits very high sensitivity and specificity in detecting the majority of solid organ injuries, but unfortunately misses up to 15% of small bowel and mesenteric injuries as well as some acute pancreatic injuries [3,4]. Protocols including a short delay between intravenous contrast administration and actual CT imaging aim to improve diagnostic accuracy in blunt abdominal trauma [5]. Although patients with solid organ injury may benefit from this strategy, patients with free fluid as only visible intraabdominal pathology or patients with suspected viscus injury did not profit from this diagnostic strategy.

Various authors have evaluated the benefits (or disadvantages) of the addition of contrast agent for CT scanning. Older studies usually base their protocols on conventional or single-detector row helical CT scan with use of oral and intravenous contrast. Although relatively rare and not always easy to detect [6], extravasation of oral contrast is highly specific for damage to the bowel and nearly always results in further surgical exploration. Those opposing the use of oral contrast argue the potential delay in patient care and the risk of aspiration [7], which although relatively uncommon [8], can end disastrous for the patient. Newer studies using (multi-detector) CT scanners in which oral contrast was omitted show comparable results [9,10], indicating, that administration of oral contrast can be avoided.

In centers where a CT scan is not available or limited to office hours, frequent re-evaluation of the patient's condition, repeated sonography and DPL remain the cornerstones of the diagnostic work-up of abdominal trauma. In the setting where clinical evaluation alone is relied on to determine whether or not a patient requires surgery, negative laparotomy rates may be up to 40% [11]. In centers where a positive DPL is regarded as the gold standard when deciding on an intervention, diagnostic laparoscopies or laparotomies are performed routinely. The downside of this strategy is a potentially high number of unnecessary or non-therapeutic operations [12], its unreliability in detecting retroperitoneal injuries [13] and, if performed too soon after initial trauma, can miss intestinal perforation [14].

Where CT scanning is readily available, up to 85% of abdominal solid organ injuries are treated conservatively [15]. Fortunately, the majority of these patients have direct or indirect signs of organ damage, which guide the trauma surgeon through the jungle of different decision pathways [16]. Even in patients with gun shot wounds to the abdomen, for whom operative management has, until recently, been viewed as mandatory, abdominal CT scanning has proven itself to be a safe and useful method for selecting patients for non-operative treatment [17-19]. In general, there is no doubt that CT is extremely useful in patients with suspected abdominal solid organ injuries. Nowadays, a trauma surgeon's life without CT is inconceivable, especially for the new generation, trained in an era when CT has always been available [20].

But what should be done if the "almighty CT scanner" does not provide us with a conclusive answer to our questions? One of the most difficult diagnostic challenges is the presence of free fluid in the abdomen without evidence of solid organ injury. In order to find an answer to our question as to what should be done for patients in this setting, we searched Pubmed for "free fluid (without) solid organ injury".

The literature on this topic, which cites more than 50 publications in English alone - mostly retrospective reviews of patient data - gives us an abundance of options to deal with this dilemma. Recommendations vary from sole observation with serial abdominal examinations, to further evaluation with additional radiological studies, DPL and/or surgical intervention [21-28].

A major limitation of all the published studies is the inclusion of only a small number of full thickness hollow organ injuries, which can be the source of the free abdominal fluid. In some studies, the number of patients with blunt (abdominal) trauma presenting with free fluid but without obvious organ injury is as low as 0.5%, especially if the study population has a high ratio of male patients, which is often the case in trauma patients [22]. The low incidence of such injuries may be one reason why no randomized prospective controlled trials have been performed.

One of the largest systematic reviews, conducted by Rodriguez and co-workers, found 10 articles in which isolated free abdominal fluid was seen without organ injury [21]. The study included 463 patients out of a total of 16000 (2.8%) with signs of free intra-abdominal fluid without obvious solid organ injury who had received a CT scan for blunt abdominal trauma. A therapeutic laparotomy was performed in only 122 patients and the authors concluded that laparotomy is not warranted if the patient is alert and can be monitored with repeated physical examination.

Although the preferred surgical access still is mainly via quick and easy laparotomy, diagnostic laparoscopy, especially in the more stable patient, provides all the advantages of minimally invasive elective surgery. A recent study from Cherkasov and coauthors, although retrospective, was able to demonstrate the advantages of the less traumatic, safe and feasible technique of video-assisted minimally invasive surgery [29].

In a more recent single centre review of 2651 trauma admissions, 14 (0.5%) patients had free intraabdominal fluid without solid organ injury in the initial CT scan [22]. Eleven of these 14 patients underwent therapeutic laparotomy based on the presence of hypotension, peritoneal irritation or additional findings on CT associated with non-solid organ injury. In their discussion, Yegiants et al. stressed that the decision on whether to operate or not is made too often by solely relying on the surgeon's personal experience - with the amount of free fluid detected rarely playing a role [22].

Some authors suggest that traces of free fluid in the pelvis, even so for male patients, with no other signs of injury are not associated with significant intra-abdominal injury and can be safely managed non-operatively [24]. The presence of more than "just a trace" is rare, but is a significant indicator of intra-abdominal injury [24].

Others, like Malhotra and colleagues, concentrate their evaluation more on the number of additional positive findings, rather than the actual amount of free fluid, which can be used to increase the accuracy of the CT scan [27]. In a series of 8112 scans, they found only seven patients with false negative scans. In addition to free fluid signs of a pneumoperitoneum, mesenteric streaking, thickened bowel wall and extravasation of contrast material were associated with hollow viscus injuries. Once again, the small number of patients included with free abdominal fluid without solid organ injury limits the conclusions of this study.

Whilst surgically important bowel and/or mesenteric injuries are usually accurately revealed using multi-detector CT imaging [30], these injuries are not always associated with extraluminal contrast material, abrupt termination of mesenteric vessels or even contrast extravasation from the mesenteric vessels. In such a setting even larger injuries can be initially missed. Unfortunately, missed intra-abdominal hollow organ injuries have a high morbidity, with mortality reaching 31% if undiagnosed for more than 24 hours [31-33].

Even improvements in diagnostic equipment, such as contrast-enhanced ultrasound or new generation multi-detector CT scanners, have not been able to prove their efficacy yet. Both ultrasound and CT-based diagnostic algorithms have been proposed, but unfortunately hollow viscus injuries can be missed by both radiological examinations. Neither repeated clinical follow-up nor repetitive CT scan imaging revealed hollow viscus injury in the case series of Permentier et al. [33]. The authors were disappointed by the possibilities of modern imaging technology and suggest traditional DPL, accompanied by the determination of the cell count ratio, to reveal any injuries at an early stage. In hemodynamically stable patients, DPL should incorporate analysis of the cell count ratio, amylase and alkaline phosphatase levels and the presence of food fibers or bile. In hemodynamically unstable patients, explorative surgery should be carried out, as this usually suggests damage to vascular structures rather than rupture of a hollow viscus [33]. Otomo et al. and Hennemann et al. have tried to refine the criteria for positive DPL [34,35]. The ratio of white blood cells (WBC) to red blood cells (RBC) can be used, where a ratio of WBC:RBC 1: 150 is regarded as being a positive finding [34]. Hennemann corrects the WBC in the lavage fluid for the WBC in the peripheral blood [35]. Unfortunately, both studies lack the statistical evidence required to make DPL a valid tool in the setting of abdominal trauma with evidence of free fluid and without obvious solid organ injury. Another hailed imaging tool, ultrasound, has also failed as the diagnostic method of choice. Hollow viscus injuries do not tend to bleed extensively so unless large volumes of fluid have leaked out of, for example due to a larger perforation of the bowel, positive predictive values remain very low (38%) [36]. CT has proven to be equally unreliable in this setting with a sensitivity ranging between 0% and 85% [37]. Even combinations of additional positive predictive signs, such as the presence of a pneumoperitoneum and visceral organ wall thickening, are not able to increase CT sensitivity and specificity beyond 80%. The only obvious sign of a hollow organ perforation remains extravasation of oral contrast [38,39].

In alert and non-comatose patient, physical examination (presence of peritonitis) is the method of choice to rule out significant abdominal injury. However, signs of peritonitis may take hours before becoming clinically evident, which is an important downside of this strategy. If the patient is intubated, intoxicated or suffers from impaired neurological function (e.g. tetraplegia), any clinical examination loses its value and the decision to carry out a surgical intervention (or not) based solely on clinical findings becomes unreliable [40,41]. In his series of 90 patients with free intraabdominal fluid but without solid organ injury, Livingston showed that 19% of patients without abdominal tenderness actually had an abdominal injury [40]. One indirect sign, which seems to be associated with hollow organ injury (if free fluid without solid organ injury is found) are seat belt marks, which increase the likelihood of an abdominal injury 2- to 4-fold [42,43].

In a study by Chandler et al., 117 victims involved in a motor vehicle accident were evaluated for the use of seatbelts and the presence or absence of a seatbelt mark [42]. 14 of 117 (12%) patients had a seatbelt sign. Three of these patients (21%) had a small bowel perforation. In contrast, in the group of 103 patients without a seatbelt sign, only two (1.9%) patients had small intestine perforation. The authors concluded that the presence of a seatbelt mark is associated with an increased likelihood of abdominal and especially intestinal injuries and mandates a heightened level of suspicion [42].

In an older study, Appleby and co-workers investigated 36 patients with seatbelt marks who underwent laparotomy after a motor vehicle accident [43]. A high incidence of gastrointestinal injuries (67%) was noted in this group. But again, the small sample size limits the value of this study [43].

## Summary

**Figure 1:**
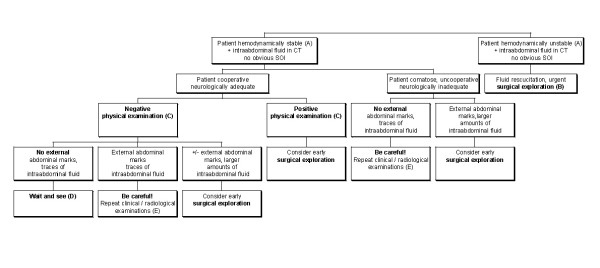
**The algorithm summarizes a possible plan of action for patients who have sustained blunt abdominal injury with suspected intraabdominal injury other than solid organ damage**. **(A) **Patient can be stabilized with adequate fluid management. **(B) **Depending on local skill and availability of theatre resources, laparoscopy is the preferred method of choice. **(C) **Positive/negative physical examination: refers to clinical signs of peritonitis. **(D) **Small amounts of abdominal fluid, especially in the female patient, may be physiological. Even in the absence of any clinical signs and abdominal marks, the patient should be evaluated on a regular basis, as some injuries require a certain time to become clinically manifest. **(E) **The risk of intraabdominal injury is greatly increased if abdominal marks (such as seat belt marks) are present. Special care needs to be taken so as not to miss any changes in patient presentation.

In accordance with the literature and to the best of our knowledge, we suggest an algorithm (see figure 1 for details), which involves asking oneself a few simple questions at the time-point of initial patient evaluation.

1. How hemodynamically stable is the patient?

2. How much fluid is present and where is the fluid located?

3. How alert is the patient and how reliable is the clinical examination?

4. Are there seat belt marks or other abdominal wall marks indicating direct trauma to the abdomen?

5. Have we been able to read the CT scan correctly?

In the hemodynamically unstable patient, there is no place for any academic discussions and the source of bleeding should be sought aggressively. If there is evidence of free intra-abdominal fluid and the patient is stable and not requiring urgent and immediate surgical exploration of the abdomen, laparoscopy may be the diagnostic method of choice.

The technique chosen (laparotomy versus laparoscopy) obviously depends on the surgeon's experience and the overall hospital culture. In Europe laparoscopy is considered the surgical technique of choice, although it has it downsides [44]. It is expensive, stretching the theatre after-hour resources to the limit and is unreliable in the hands of the inexperienced surgeon. If used correctly though, it provides a less traumatic option and reduces possible complications associated with a large incision.

**Figure 2:**
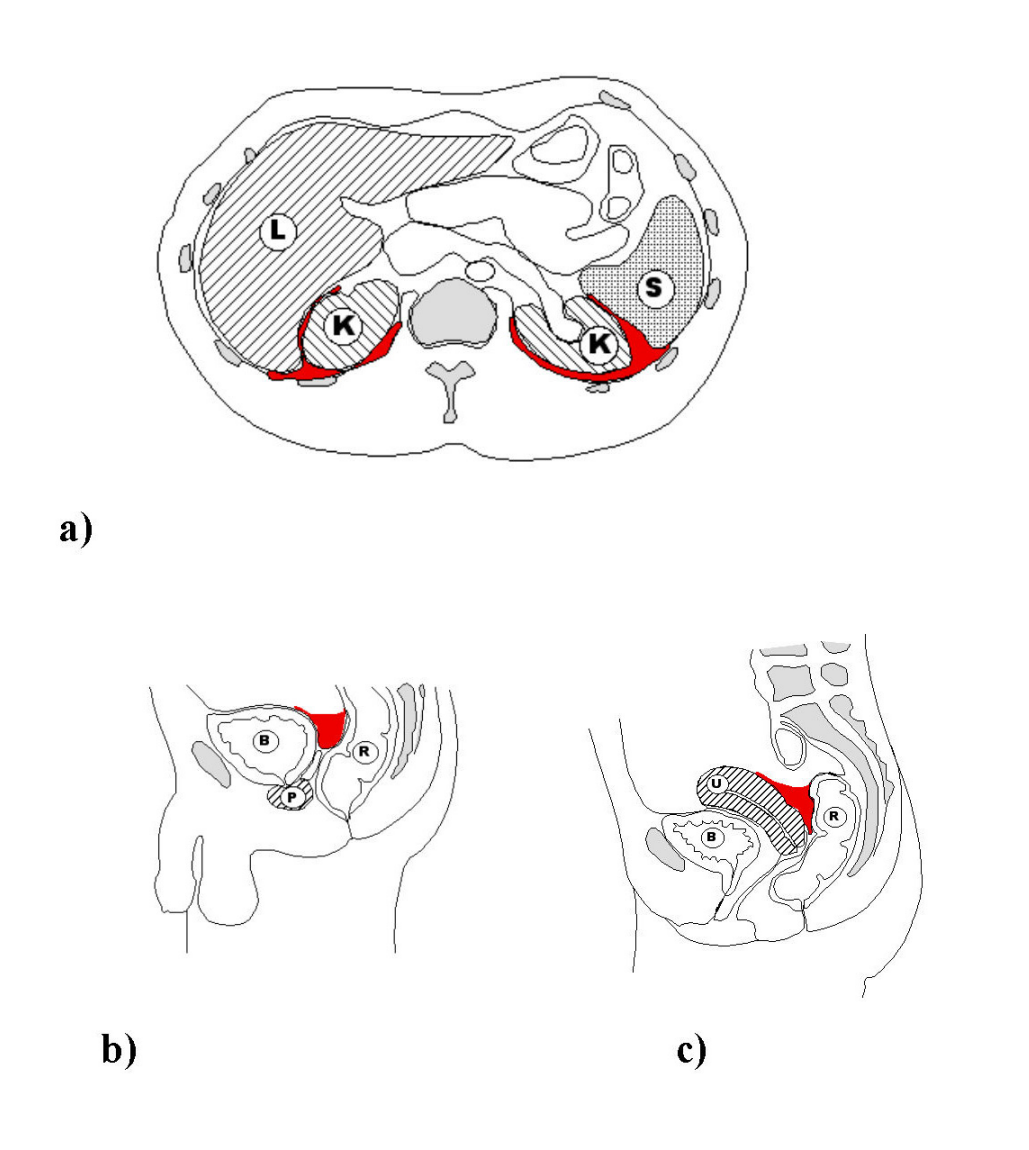
**a-c. a) Schematic drawing of a transverse section of the abdominal cavity, depicting fluid (in red) in the Morison pouch on the right, between kidney (K) and the liver (L) and on the left free fluid between the left kidney and the spleen (S)**. b) Male anatomy, sagittal plane, with free fluid in the pouch of Douglas (in red) between the bladder (B) and the rectum (R). Prostate gland (P). c) Female anatomy, sagittal plane, with free fluid in the pouch of Douglas (in red) between the uterus (U) and the rectum (R). Bladder (B).

**Figure 3:**
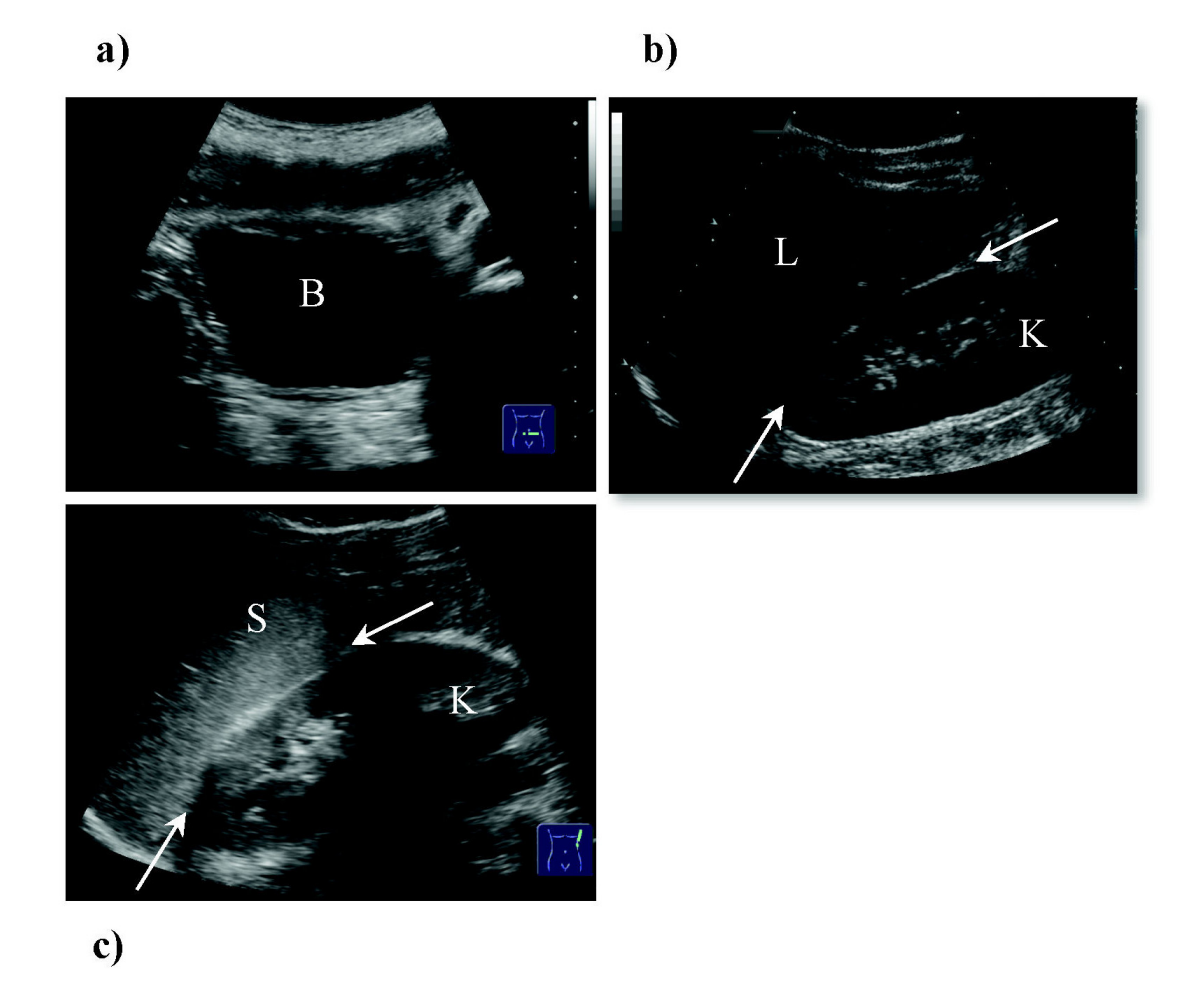
**a-c. a) Rectouterine pouch (females) or rectovesical excavation (males), also known as the pouch of Douglas**. In females a tiny amount of free fluid is physiological, particularly after ovulation. B = Bladder. b) Hepatorenal recess (L = liver, K = right kidney), also known as Morison's pouch. Already small amounts of fluid can be detected in this potential space and are an indication of free intraabdominal fluid. c) Space between the spleen (S) and the left kidney (K). Fluid detected in this space may be an indication of splenic trauma but is non-specific and can be due to any form of free intraabdominal collection (blood/ascites/intestinal leakage).

Whilst evaluating the patient we suggest distinguishing between a trace (minimal fluid in one region), and larger amounts of free fluid (also seen as fluid in multiple areas). According to the literature, over 70% of patients will fall into the first category, and conservative treatment of these patients is thought to be safe in the vast majority of cases [23]. As might be expected, the pouches of Douglas and Morrison are the two most common locations for free fluid. No other inter-peritoneal location seems to be associated with organ injury [23]. See figures 2 and 3 for details.

Question number five, whether or not the CT scan has been interpreted correctly is probably the most challenging question to answer and problem to solve.

## Conclusions

A thorough literature search has made it apparent that there is no straightforward answer to the question of what to do with patients with free fluid on CT scanning but without signs of organ injury. All studies, whether they are prospective or retrospective, lack the statistical power to provide a definite answer. Furthermore, the studies are difficult to compare, as there are significant differences in imaging equipment, laboratory workup and surgical experience. The majority of the studies investigated inhomogeneous groups of patients or had methodological or statistical problems.

Fortunately this type of injury is very rare. This in turn, however means that exposure to such cases in the course of one's career is infrequent, making it difficult to rely on general experience alone to correctly diagnose and adequately treat such injuries.

## Competing interests

The authors declare that they have no competing interests.

## Authors' contributions

VB designed the study and drafted the manuscript, HZ coordinated and helped to draft the manuscript, MB helped conceive the study and contributed to the revisions, VJ helped revise the manuscript and designed Figures 2a-c. AE conceived of the study, participated in the design of the study and helped to draft the manuscript. All authors read and approved the final manuscript.
